# *miR-139-5p* inhibits isoproterenol-induced cardiac hypertrophy by targetting c-Jun

**DOI:** 10.1042/BSR20171430

**Published:** 2018-03-09

**Authors:** Su Ming, Wang Shui-yun, Qiu Wei, Li Jian-hui, Hui Ru-tai, Song Lei, Jia Mei, Wang Hui, Wang Ji-zheng

**Affiliations:** 1 Department of Clinical Laboratory, Peking University People’s Hospital, Beijing, People’s Republic of China; 2 State Key Laboratory of Cardiovascular Disease, Fuwai Hospital, National Center for Cardiovascular Disease, Chinese Academy of Medical Sciences and Peking Union Medical College, Beijing, People’s Republic of China; 3 Department of Cardiovascular Surgery, Fuwai Hospital, National Center for Cardiovascular Diseases, Chinese Academy of Medical Sciences and Peking Union Medical College, Beijing, People’s Republic of China; 4 Department of Urology, Beijing Friendship Hospital, Capital Medical University, Beijing, People’s Republic of China; 5 National Research Institute for Family Planning, Peking Union Medical College, Beijing, People’s Republic of China

**Keywords:** c-Jun, hypertrophic cardiomyopathy, isoproterenol, miR-139-5p

## Abstract

Hypertrophic cardiomyopathy (HCM) is a serious monogenic disease characterized by cardiac hypertrophy, fibrosis, sudden cardiac death, and heart failure. Previously, we identified that *miR-139-5p* was down-regulated in HCM patients. However, the regulatory effects of *miR-139-5p* remain unclear. Thus, we investigated the role of *miR-139-5p* in the regulation of cardiac hypertrophy. The expression of *miR-139-5p* in left ventricular tissues in HCM patients and mice subjected to transverse aortic constriction (TAC) was significantly down-regulated. Knockdown of *miR-139-5p* expression in neonatal rat cardiomyocytes (NRCMs) induced cardiomyocyte enlargement and increased atrial natriuretic polypeptide (ANP) expression. Overexpression of *miR-139-5p* antagonized isoproterenol (ISO)-induced cardiomyocyte enlargement and ANP/brain natriuretic peptide (BNP) up-regulation. More importantly, we found that c-Jun expression was inhibited by *miR-139-5p* in NRCMs. Knockdown of c-Jun expression significantly attenuated cardiac hypertrophy induced by *miR-139-5p* deprivation. Our data indicated that *miR-139-5p* was down-regulated in the hearts of HCM patients and that it inhibited cardiac hypertrophy by targetting c-Jun expression.

Hypertrophic cardiomyopathy (HCM) is a monogenic disease with an estimated prevalence of 0.2% in the population [1]. This disease is characterized by cardiac hypertrophy, fibrosis, sudden cardiac death, and heart failure [2,3]. Although it has been revealed that HCM is mostly caused by mutations in sarcomere genes [4–6], the regulatory mechanisms of cardiac hypertrophy in HCM are not fully understood.

MicroRNAs (miRs) are intracellular small, non-coding RNAs that mainly down-regulate specific target genes by binding to the 3′-UTR of corresponding mRNAs [7]. Accumulating studies have revealed that miRs play important roles in regulating cardiac remodeling in HCM, thereby providing potential therapeutic targets for this disease [8–11]. By using microarray analyses, we previously screened for miRs in left ventricular tissues that were differentially expressed between HCM patients and healthy donors [9]. We identified that *miR-139-5p* was one of the most down-regulated miRs in the hearts of HCM patients [9]. However, whether *miR-139-5p* participates in the regulation of cardiac hypertrophy remains unclear.

The β-adrenergic receptor (β-AR) plays an important role in the regulation of cardiac excitation–contraction; however, hyperactivation of β-AR leads to cardiac remodeling [12,13]. The stimulation of β-AR activates the downstream activating protein-1 (AP-1), which then transmits signaling to induce cardiac hypertrophy [14,15]. AP-1 is a transcription factor consisting of a group of immediate early genes, such as *c-Jun* and *c-Fos*. They form homo- or heterodimers to regulate the expression of their target genes. By using online bioinformative assays (www.targetscan.org), we predicted that c-Jun might be a target of *miR-139-5p* in cardiomyocytes, which might participate in the regulation of cardiac hypertrophy. Therefore, we investigated this aspect in the present study. We found that isoproterenol (ISO), a potent β-AR agonist, strongly induced the expression of c-Jun, whereas exogenous addition of *miR-139-5p* mimic antagonized its expression and attenuated ISO-induced cardiac hypertrophy.

## Materials and methods

### Ethics statement

All protocols pertaining to human subjects were approved by the Ethics Committee of Fuwai Hospital (Beijing, China) in accordance with the Declaration of Helsinki. Written informed consent was obtained from all the participants. Left ventricular tissues were obtained as previously described [9]. All the protocols pertaining to animals were approved by the Ethics Committee for Animal Study of Fuwai Hospital (Beijing, China) (approval number: 0088-R/M-300/200-GZ(X)).

### Transverse aortic constriction operation

Transverse aortic constriction (TAC) operation in mice was performed as previously described [16]. In brief, 6–8 weeks old male C57BL/6 mice were weighed and anesthetized with 2.5% avertin (0.018  ml/g) through intraperitoneal injection. The transverse aorta was ligated for TAC operation. Sham-operated mice served as controls. All mice were killed at 4 weeks post operation, and the hearts were harvested for *miR-139-5p* expression analysis.

### Cell culture

Primary neonatal rat cardiomyocytes (NRCMs) were cultured as previously described [17]. In brief, ventricles from neonatal Wistar rats were isolated and cut into small pieces. The tissues were washed with PBS and digested with 0.06% collagenase II (Worthington Biochemical Corporation, Lakewood, NJ, U.S.A.) at 37°C. The cell suspension was centrifuged and resuspended with Dulbecco’s modified Eagle’s medium (DMEM) containing 10% FBS. For differential adhesion, the cells were maintained in an atmosphere with 5% CO_2_ at 37°C for 90 min. The supernatants were resuspended with complete DMEM containing 0.1 mM bromodeoxyuridine, seeded in corresponding plates and routinely cultured for 24 h. Then, the medium was replaced with DMEM containing 1% FBS for experiments.

### Cell transfection and treatment

NRCMs were transiently transfected with *miR-139-5p* mimics or inhibitors (GenePharma, Suzhou, China) at a final concentration of 100 nM. For the knockdown of c-Jun expression, a predesigned specific siRNA was used (Silencer^®^ Select, s127979, Thermo Fisher, Carlsbad, CA, U.S.A.). All the above small RNAs were transfected by using Lipofectamine^®^ RNAiMAX Reagent (Thermo Fisher) according to the manufacturer’s protocol. For inducing cardiac hypertrophy *in vitro*, NRCMs were treated with 100 nmol/l ISO for 24 h [18].

### Determination of cell surface area

NRCMs (5 × 10^5^ cells/ml) were seeded in 24-well plates and routinely cultured. The cells were fixed with 4% paraformaldehyde and permeabilized with 1% Triton X-100 in PBS for 5 min. The cells were then washed with PBS and stained with Texas Red-Phalloidin (Thermo Fisher) for 30 min at room temperature. The nuclei were stained with 0.1 μg/ml DAPI for 5 min. The cells were visualized with a fluorescence microscope, and the cell surface area was measured by analyzing 30 NRCMs from at least five random fields with ImagePro Plus 6.0 Software (Media Cybernetics, Bethesda, MD, U.S.A.).

### Quantitative real-time PCR analysis

Total RNA was extracted by using TRIzol reagent (Thermo Fisher), quantitated, and normalized. To analyze the expression of *miR-139-5p*, a specific primer containing a stem loop structure was used for cDNA synthesis with a cDNA synthesis kit (Takara, Dalian, China): 5′-CTCAACTGGTGTCGTGGAGTCGGCAATTCAGTTGAGACTGGAGA-3′. The expression of *miR-139-5p* was determined using the following primers: forward, 5′-ACACTCCAGCTGGGTCTACAGTGCACGTGTC-3′ and reverse, 5′-TGGTGTCGTGGAGTCG-3′, and normalized to U6 expression determined with the following primers: forward, 5′-CTCGCTTCGGCAGCACA-3′ and reverse, 5′-AACGCTTCACGAATTTGCGT-3′.

For the routine quantitation of mRNAs, cDNA was synthesized with a cDNA Synthesis Kit (Takara). The following specific primers for real-time PCR were used: atrial natriuretic polypeptide (ANP) forward, 5′-GGGCTCCTTCTCCATCAC-3′ and reverse, 5′-CGGCATCTTCTCCTCCAG-3′; brain natriuretic peptide (BNP) forward, 5′-AGAACAATCCACGATGCAGAAG-3′ and reverse, 5′-AAACAACCTCAGCCCGTCACA-3′; and 18S rRNA forward, 5′-CTTAGAGGGACAAGTGGCG-3′ and reverse, 5′-GGACATCTAAGGGCATCACA-3′. The relative expression of target genes was determined by using the ΔΔ*C*_t_ (cycle threshold) method.

### Western blotting

Total protein was extracted with RIPA lysis buffer, quantitated, and normalized. Approximately 20 μg protein from each sample was separated using SDS/PAGE and blotted on to a nitrocellulose membrane. The membrane was blocked with 5% BSA at room temperature for 1 h and then incubated with the following primary antibodies at 4°C overnight: rabbit anti-Akt, anti-p-Akt, anti-β-catenin, anti-GAPDH (Cell Signaling Technology, Beverly, MA, U.S.A.), anti-c-Jun, anti-IGF-1 receptor (anti-IGF-1R) (Santa Cruz Technology, Dallas, TX, U.S.A.), anti-myocardin (anti-MyoCD) (Sigma, St. Louis, MO, U.S.A.), and anti-Wnt1 (Abcam, Cambridge, MA, U.S.A.). Then, the membrane was washed three times with TBS Tween-20 (TBS-T) and incubated with goat anti-rabbit secondary antibody (Cell Signaling Technology) at room temperature for 1 h. The bands were visualized and analyzed using Quantity One software V 4.6.2 (Bio–Rad, Hercules, CA, U.S.A.).

### Statistical analysis

Statistical analyses were performed with IBM SPSS 19.0 Software (SPSS, Inc., Chicago, IL, U.S.A.). Two-tailed Student’s *t* test or ANOVA was used to determine the differences. The data are expressed as the mean ± S.E.M., and *P*<0.05 was considered statistically significant.

## Results

### *miR-139-5p* is down-regulated in hypertrophic hearts

**Figure 1:**
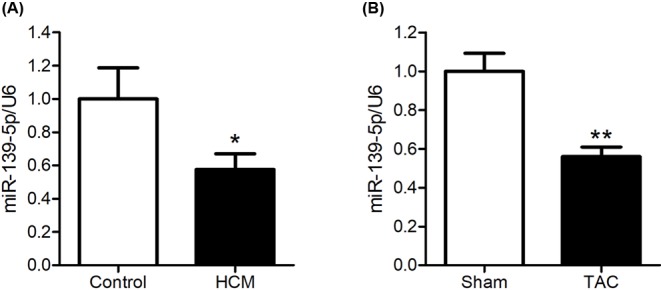
*miR-139-5p* is down-regulated in hypertrophic hearts (**A**) Expression of *miR-139-5p* in 16 HCM patients and 8 healthy donors (Control). (**B**) Expression of *miR-139-5p* in mice subjected to TAC (*n*=8); sham-operated mice were used as controls (*n*=8). Real-time PCR assay was performed; and U6 was employed as an internal control. Data represent the mean ± S.E.M. *P<0.05 and **P<0.01 compared with the control group.

To validate whether *miR-139-5p* is down-regulated in hypertrophic hearts, we detected its expression in left ventricular tissues from 16 HCM patients and 8 healthy donors. We found that the expression of *miR-139-5p* in left ventricular tissues of HCM patients was down-regulated to 57.6% of that in the healthy donors (*P*<0.05, power: 0.505) (Figure 1A). We then performed TAC operation to induce cardiac hypertrophy in mice. Consistently, the expression of *miR-139-5p* in the hearts of TAC-operated mice was significantly down-regulated compared with that in sham-operated mice, indicating that *miR-139-5p* is down-regulated in hypertrophic hearts (*P*<0.01, power: 0.765) (Figure 1B).

### *miR-139-5p* is required for maintaining normal cell size in cardiomyocytes

**Figure 2:**
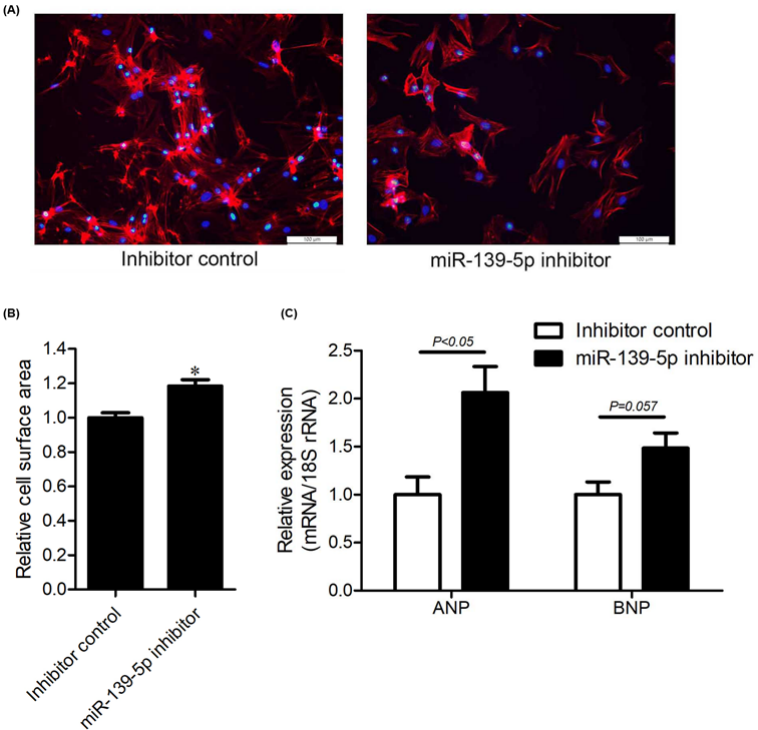
Knockdown of *miR-139-5p* expression induces cardiac hypertrophy. (**A–C**) NRCMs were transfected with *miR-139-5p* inhibitor or the inhibitor control RNA for 48 h. Intracellular F-actin was stained with Texas Red-Phalloidin, and nuclei were stained with DAPI; scale bars: 100 μm (A). Cell surface area was quantitated with ImagePro Plus 6.0 from at least 30 cells in three independent experiments. Data are expressed as the mean ± S.E.M. *P<0.05 compared with the controls (B). Expression of ANP and BNP were quantitated by using real-time PCR. 18S rRNA was used as the loading control. Relative expression was indicated as the mean ± S.E.M. (*n*=4), and *P*-values are shown as indicated (C).

To investigate the role of *miR-139-5p* in cardiac hypertrophy, we knocked down its expression in NRCMs by transfecting a specific miR inhibitor. The levels of *miR-139-5p* were down-regulated to 25.4% of that in control NRCMs at 48 h post transfection (Figure 2A). We then measured changes in cell surface area by staining NRCMs with Texas Red-conjugated Phalloidin. We found that the cell surface area was significantly increased in *miR-139-5p*-deprived NRCMs (Figure 2B). ANP and BNP are two known biomarkers of cardiac hypertrophy. The knockdown of *miR-139-5p* induced a 2.06-fold increase in ANP expression (*P*<0.05); however, the expression of BNP was unchanged (Figure 2C). These results indicated that *miR-139-5p* is required to maintain normal cell size in cardiomyocytes.

### Overexpression of *miR-139-5p* attenuates ISO-induced cardiac hypertrophy

**Figure 3:**
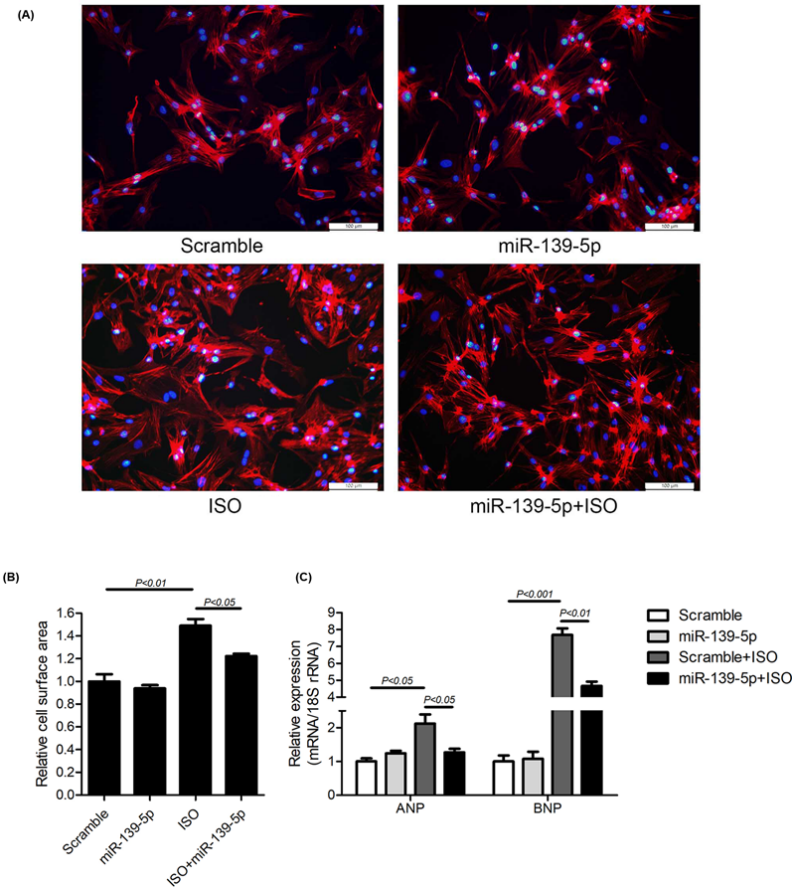
Overexpression of *miR-139-5p* attenuates ISO-induced cardiac hypertrophy (**A**–**C**) NRCMs were transfected with the *miR-139-5p* mimic or scrambled control small RNAs for 48 h, followed by ISO treatment for 24 h. Intracellular F-actin and nuclei were stained with Texas Red-Phalloidin and DAPI, respectively. Scale bars: 100 μm (A). Cell surface area was measured using ImagePro Plus 6.0 software from at least 30 cells in three independent experiments (B). Expression of ANP and BNP were quantitated by using real-time PCR and normalized to the level of 18S rRNA (*n*=3 per group) (C). Data are indicated as the mean ± S.E.M., and *P*-values are shown as indicated.

Since *miR-139-5p* participates in the regulation of cell size in cardiomyocytes, we wanted to explore whether the exogenous administration of *miR-139-5p* is able to antagonize cardiac hypertrophy. ISO is a β-AR agonist that is widely used to induce cardiac hypertrophy. Treating NRCMs with 100 nmol/l ISO for 24 h induced a significant increase in cell size (Figure 3A,B). Pre-transfection of NRCMs with the *miR-139-5p* mimic for 48 h attenuated ISO-induced cellular enlargement (Figure 3A,B). Consistently, the expression of ANP and BNP was up-regulated by ISO treatment by 2.11- and 7.68-fold, respectively (Figure 3C), whereas the transfection of NRCMs with the *miR-139-5p* mimic attenuated the ISO-induced increase in ANP and BNP expression (Figure 3C). In addition, *miR-139-5p* did not affect cell surface area or ANP/BNP levels under basal conditions (Figure 3A–C). These data indicated that *miR-139-5p* attenuates ISO-induced cardiac hypertrophy *in vitro*.

### *miR-139-5p* down-regulates c-Jun expression in ISO-induced cardiac hypertrophy

**Figure 4:**
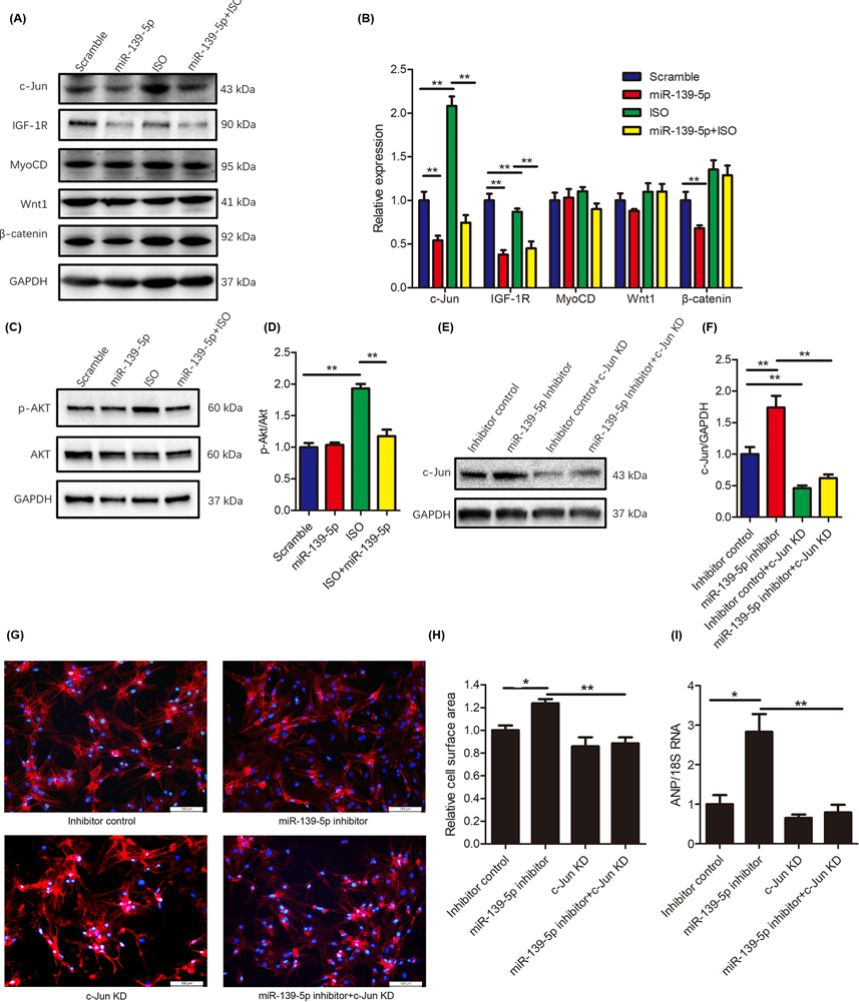
*miR-139-5p* targets c-Jun expression in cardiomyocytes (**A**–**D**) NRCMs were treated as indicated. Levels of c-Jun, IGF-1R, MyoCD, Wnt1, and β-catenin (A) and the phosphorylation status of Akt (B) were detected using Western blotting. The relative expression intensity (B,D) was obtained from three independent experiments were performed. (**E**–**I**) siRNAs for knocking down (KD) c-Jun expression were co-transfected into to NRCMs with the *miR-139-5p* inhibitor or the inhibitor control RNA for 48 h. Expression of c-Jun was determined using Western blotting (E) and the relative expression of c-Jun was quantitated (F). F-actin and nuclei were stained with Texas Red-Phalloidin and DAPI, respectively. Scale bars: 100 μm (G). Cell surface area was quantitated from at least 30 cells in three independent experiments (H). Expression of ANP was quantitated by using real-time PCR and normalized to the level of 18S rRNA (*n*=3 per group) (I). Data are indicated as the mean ± S.E.M., *P<0.05 and **P<0.01 compared between the indicated groups.

miRs specifically bind to the 3′-UTR of its target mRNAs to regulate their expression. By using TargetScan (http://www.targetscan.org/vert_71/), an online tool for predicting miR target genes, we identified c-Jun, IGF-1R, MyoCD, Wnt1, and β-catenin as candidate target genes of *miR-139-5p*. We transfected NRCMs with the *miR-139-5p* mimic for 48 h and found that the levels of c-Jun, IGF-1R, and β-catenin were all down-regulated, whereas the levels of MyoCD and Wnt1 were unaffected (Figure 4A,B). Interestingly, *miR-139-5p* overexpression inhibited the ISO-induced up-regulation of c-Jun expression (Figure 4A,B). In contrast, the knockdown of endogenous *miR-139-5p* increased the level of c-Jun (Figure 4E,F). These results indicated that *miR-139-5p* inhibits the expression of c-Jun in cardiomyocytes and thus might play an important role in the regulation of cardiac hypertrophy.

The expression of IGF-1R was down-regulated by *miR-139-5p* in NRCMs, although ISO alone slightly down-regulated IGF-1R expression (Figure 4A,B). The IGF-1R signaling pathway has been reported to regulate cardiac hypertrophy via PI3K/Akt [19]. As expected, *miR-139-5p* suppressed ISO-induced hyperphosphorylation of Akt in NRCMs, indicating that the IGF-1R/Akt pathway might also contribute to the antihypertrophic effects of *miR-139-5p* (Figure 4C,D). Wnt1/β-catenin has been reported to be regulated by *miR-139-5p* [20]. We observed that *miR-139-5p* indeed down-regulated β-catenin expression under basal conditions, but it failed to inhibit Wnt1 or β-catenin expression in response to ISO stimulation (Figure 4A,B).

### Inhibition of c-Jun activation suppresses *miR-139-5p* knockdown-induced cardiac hypertrophy

As the expression of c-Jun was found to be regulated by *miR-139-5p* in NRCMs, we proposed that the up-regulation of c-Jun might be required in *miR-139-5p* deprivation-induced cardiac hypertrophy. To investigate this aspect, we knocked down c-Jun expression by transfecting c-Jun-specific siRNAs into *miR-139-5p*-deprived NRCMs. The knockdown of *miR-139-5p* expression in NRCMs significantly increase c-Jun levels, whereas the knockdown of c-Jun expression prevented this increase (Figure 4E,F). As expected, the increase in cell surface area and the up-regulation of ANP expression induced by *miR-139-5p* deprivation were restored by c-Jun knockdown (Figure 4G–I). These results indicated that c-Jun is indispensable in *miR-139-5p* deprivation-mediated cardiac hypertrophy *in vitro*.

## Discussion

Pathological cardiac remodeling in HCM is associated with the deregulated expression of many intracellular miRs [8–11]. In the present study, we reported that *miR-139-5p* was down-regulated in heart tissues of HCM patients. The knockdown of *miR-139-5p* expression in NRCMs induced cardiac hypertrophy, whereas the exogenous overexpression of *miR-139-5p* antagonized ISO-induced cardiac hypertrophy. These findings indicated that *miR-139-5p* acts an anti-hypertrophic miR in the heart.

HCM is a monogenic myocardial disease, which is characterized by cardiac hypertrophy, sarcomere disarrangement, cardiac fibrosis, heart failure, and cardiac sudden death [2]. By using microarray analysis, we found that *miR-139-5p* was significantly down-regulated in the left ventricular tissues of HCM patients. Moreover, the expression of *miR-139-5p* was down-regulated in the hearts of TAC-operated mice, and knockdown of *miR-139-5p* in NRCMs induced cardiomyocyte enlargement. These data indicated that *miR-139-5p* is indispensable in normal cardiomyocytes.

The β-AR signaling pathway, which is classically activated by catecholamines, is one of the well-known pathways in the induction of cardiac hypertrophy [12,13]. The activation of β-AR regulates cardiac excitation–contraction and leads to the increase in heart rate and cardiomyocyte contractility [21–23]. The hyperactivation of β-AR results in cardiac hypertrophy, fibrosis, senescence, inflammation, and cardiomyocyte apoptosis and necrosis [24–29]. The treatment of NRCMs with ISO caused cardiomyocyte enlargement and increased the expression of ANP and BNP. We observed that *miR-139-5p* overexpression attenuated ISO-induced cardiac hypertrophy, which indicated that *miR-139-5p* plays an antihypertrophic role in the heart. It has been reported that the stimulation of β-AR can induce the activation of AP-1, which is associated with the activation of the CaMKII, ERK, and JNK pathways [14,15]. AP-1 acts as a transcription factor for a group of immediate early genes, including c-Jun, c-Fos, JunB, and JunD. These proteins form homo- or heterodimers and bind to the promoters of various genes to regulate their expression, which subsequently induces cardiac hypertrophy [14,30–32]. In the present study, we observed that c-Jun expression was strongly induced by ISO-treatment, whereas *miR-139-5p* inhibited this increase. Consistently, the knockdown of c-Jun expression significantly attenuated *miR-139-5p* deprivation-induced cardiac hypertrophy. The *miR-139-5p*/c-Jun axis has been reported to form a feedback loop in human gastric cancer cells [33]. Our data indicated that the down-regulation of c-Jun is indispensable for the anti-hypertrophic effects of *miR-139-5p* in cardiomyocytes.

The activation of β-AR induced the phosphorylation of Akt/GSK3β in NRCMs, which is associated with the up-regulation of ANP expression [34]. In colitis-associated tumorigenesis, *miR-139-5p* inhibits the cross-talk between the PI3K/Akt and Wnt pathways by targetting IGF-1R [35]. Our results indicated that overexpression of *miR-139-5p* in NRCMs antagonizes ISO-induced activation of Akt. *miR-139-5p* also inhibits the expression of IGF-1R in cells both under basal and ISO-stimulated conditions, indicating that the down-regulation of IGF-1R and inhibition of Akt might be involved in preventing cardiac hypertrophy. Mi et al. [20] reported that Wnt1 and β-catenin were directly targetted by *miR-139-5p* in C2C12 cells. In our study, we observed that *miR-139-5p* indeed down-regulated β-catenin expression under basal conditions; however, it failed to antagonize the ISO-induced up-regulation of β-catenin in NRCMs. The expression of Wnt1 was not altered by *miR-139-5p* overexpression in NRCMs. These data indicated that the Wnt1/β-catenin pathway might not be associated with the antihypertrophic effects of *miR-139-5p*. In addition, we found that the predicted MyoCD was not a target of *miR-139-5p* in NRCMs, as its expression was not altered under basal or ISO-stimulated conditions.

Taken together, our study highlights that *miR-139-5p* is a novel antihypertrophic miR in cardiomyocytes. *miR-139-5p* attenuates cardiac hypertrophy possibly through the down-regulation of c-Jun expression *in vitro*. Our data indicated that the exogenous delivery of the *miR-139-5p* mimic to the heart might be a potential therapeutic strategy for pathological cardiac hypertrophy. These findings provide evidences that the *miR-139-5p*/c-Jun axis is a novel target for the prevention and treatment for cardiac remodeling. The future study will focus on the mechanism of *miR-139-5p* expression and its association with c-Jun.

## Abbreviations

ANP: atrial natriuretic polypeptide
AP-1: activating protein-1
BNP: brain natriuretic peptide
DMEM: Dulbecco’s modified Eagle’s medium
HCM: hypertrophic cardiomyopathy
IGF-1R: IGF-1 receptor
ISO: isoproterenol
miR: microRNA
MyoCD: myocardin
NRCM: neonatal rat cardiomyocyte
TAC: transverse aortic constriction
β-AR: β-adrenergic receptor
